# Spiders’ digestive system as a source of trypsin inhibitors: functional activity of a member of atracotoxin structural family

**DOI:** 10.1038/s41598-023-29576-y

**Published:** 2023-02-10

**Authors:** Oscar Bento Silva Neto, Rodrigo Valladão, Guilherme Rabelo Coelho, Renata Dias, Daniel Carvalho Pimenta, Adriana Rios Lopes

**Affiliations:** 1 Laboratory of Biochemistry, Instituto Butantan, São Paulo, 05503900 Brazil; 2 Programa Interunidades (USP, Instituto Butantan, IPT) de pós-graduação em Biotecnologia, Universidade de São Paulo, São Paulo, 05508000 Brazil; 3 Instituto de Ciências Biológicas, Universidade Federal de Goiás, Goiás, Brazil

**Keywords:** Proteomics, Proteases

## Abstract

Spiders are important predators of insects and their venoms play an essential role in prey capture. Spider venoms have several potential applications as pharmaceutical compounds and insecticides. However, transcriptomic and proteomic analyses of the digestive system (DS) of spiders show that DS is also a rich source of new peptidase inhibitor molecules. Biochemical, transcriptomic and proteomic data of crude DS extracts show the presence of molecules with peptidase inhibitor potential in the spider *Nephilingis cruentata*. Therefore, the aims of this work were to isolate and characterize molecules with trypsin inhibitory activity. The DS of fasting adult females was homogenized under acidic conditions and subjected to heat treatment. After that, samples were submitted to ion exchange batch and high-performance reverse-phase chromatography. The fractions with trypsin inhibitory activity were confirmed by mass spectrometry, identifying six molecules with inhibitory potential. The inhibitor NcTI (*Nephilingis cruentata* trypsin inhibitor) was kinetically characterized, showing a K_D_ value of 30.25 nM ± 8.13. Analysis of the tertiary structure by molecular modeling using Alpha-Fold2 indicates that the inhibitor NcTI structurally belongs to the MIT1-like atracotoxin family. This is the first time that a serine peptidase inhibitory function is attributed to this structural family and the inhibitor reactive site residue is identified. Sequence analysis indicates that these molecules may be present in the DS of other spiders and could be associated to the inactivation of prey trypsin (serine peptidase) ingested by the spiders.

## Introduction

The spider venom plays a crucial role in immobilizing the prey due to the effects of its different compounds. However, the processing of prey as a food source occurs through the use of digestive fluid, in an extra-oral digestion (EOD) process. The digestive fluid is produced at the midgut and midgut diverticula (MMD) and is rich in different classes of enzymes, such as hydrolases and other proteins involved in diverse metabolic processes^1,2^. Some of the compounds identified at the digestive fluid and MMD are peptidase inhibitors (PI), which are supposed to inhibit the prey enzymes. However, there are no detailed studies of these inhibitors synthesized in the digestive tract.

Spiders’ toxins are usually cysteine-rich peptides with different protein scaffolds, which have been classified by King and Hardy^3^ and Langenegger et al.^4^. Although their tertiary structure might be quite different, some properties due to a series of disulfide bonds, such as pH and temperature stability and resistance to peptidase hydrolysis are shared between them^4–6^. Cysteine-rich spider venom peptides adopt five main structural motifs. The inhibitory cysteine knot motif (ICK) with three disulfide bonds (C1–C4, C2–C5, C3–C6); the directed beta-hairpin loop (DDH) with two bonds (C1–C2, C2–C4); the Kunitz motif domain, frequently present in inhibitors of serine peptidases, with three bonds (C1–C6, C2–C4, C3–C5); the HAND motif identified in arthropods, with three disulfide bonds and, the motif colipase-like/MIT1 toxin-like fold with five disulfide bonds (C1–C4, C2–C5, C3–C7, C6–C9, C8–C10)^4^. Despite their similar structure scaffolds, these molecules are extremely variable in their amino acid sequence, which confers different functions and justifies their classification according to their tertiary structure and not sequence homology^7,8^.

The main trypsin and serine peptidase inhibitors are the families: serpin, Kunitz, Bowman-Birk and Kazal. Serpins are irreversible inhibitors while Kunitz, Bowman-Birk and Kazal are usually reversible tight-binding inhibitors^15^. Serpins, Kunitz and Kazal are families usually described inhibitors present in spider venom. Peptidase inhibitors are present in the venom of different species of spiders. The *Araneus ventricosus* Kunitz serine peptidase inhibitor (AvCI) is described as an inhibitor of chymotrypsin, subtilisin A and also elastase^9^. The HWTX-XI from the tarantulas *Ornithoctonus huwena* and *Ornithoctonus hainana* has a dual role as trypsin inhibitor and potassium channel blocker^9,10^. However, peptidase inhibitors have not been largely investigated in spiders' digestive systems. Pioneering investigations on digestive fluid serine peptidase inhibitors were conducted with the spider *Argiope aurantia* and evidenced their diversity and heat-stability^11^.

Although there is a lot of data on venom proteomic analysis, the evaluation of the similarities between the spiders´ MMD/digestive fluid and venom protein content was only possible recently due to the use of high-throughput techniques to perform transcriptomic and proteomic analysis of the spiders *Nephilingis cruentata* (digestive fluid and MMD)^1^; *Acanthoscurria geniculata* (digestive fluid and abdomen)^2^; *Stegodyphus mimosarum* (digestive fluid)^2^; *Loxosceles gaucho* (MMD)^1,12^. These studies revealed the presence of several molecules similar to venom toxins and peptidase inhibitors expressed and translated to protein in the digestive systems of spiders.

In order to characterize peptidase inhibitors from the spiders MMD, we isolated and characterized molecules with trypsin inhibitory activity from the MMD of *Nephilingis cruentata*.

## Results

### Purification of *Nephilingis cruentata* MMD serine peptidase inhibitors and their biochemical characteristics

**Figure 1:**
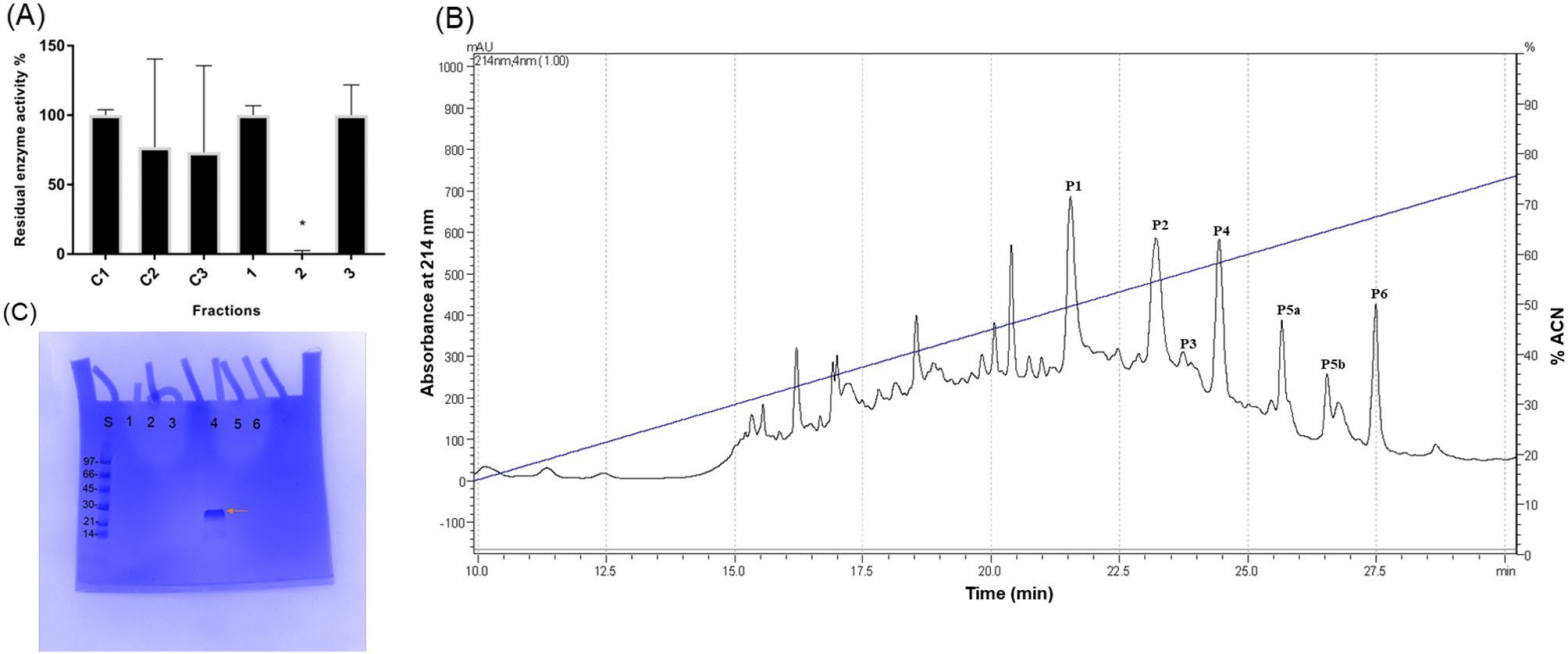
Purification of *Nephilingis cruentata* MMD inhibitors. (**A**) Bovine trypsin inhibitory assay with the eluted fractions from the ionic-exchange batch separation and respective controls. (C1): control assay of bovine trypsin with 25 mM of ammonium bicarbonate at pH 8.5; (C2): control assay of bovine trypsin 25 mM of ammonium bicarbonate containing 1 M NaCl at pH 8.5; (C3): control assay of bovine trypsin with 25 mM of ammonium bicarbonate at pH 3.5; (1): Fraction eluted with 25 mM of ammonium bicarbonate at pH 8.5; (2): Fraction eluted with 25 mM of ammonium bicarbonate with 1 M NaCl at pH 8.5; (3): Fraction eluted with 25 mM of ammonium bicarbonate at pH 3.5. Inhibitory activity was measured using bovine trypsin 12 ng/µl as enzyme source and (0.1 mM) ZFRMCA as substrate (N = 3). (**B**) Chromatographic separation profile (UV at 214 nm) of fraction 2 (inhibitory active fraction against bovine trypsin), using a C18 column. Elution was performed by a linear gradient of 5% to 90% of solution B in 35 min. (**C**) SDS-PAGE in a 4–20% polyacrylamide gel of chromatographic peaks (P1-P6). Lane S molecular mass marker (97–14 kDa); lanes respectively: 1: P1; 2: P2; 3: P3; 4: P4; 5: P5; and 6: P6. The red arrow shows a single band detected by Coomassie blue G-250R. The approximate mass for the band on line 4 is 28.78 kDa calculated by gel relative migration.

**Figure 2:**
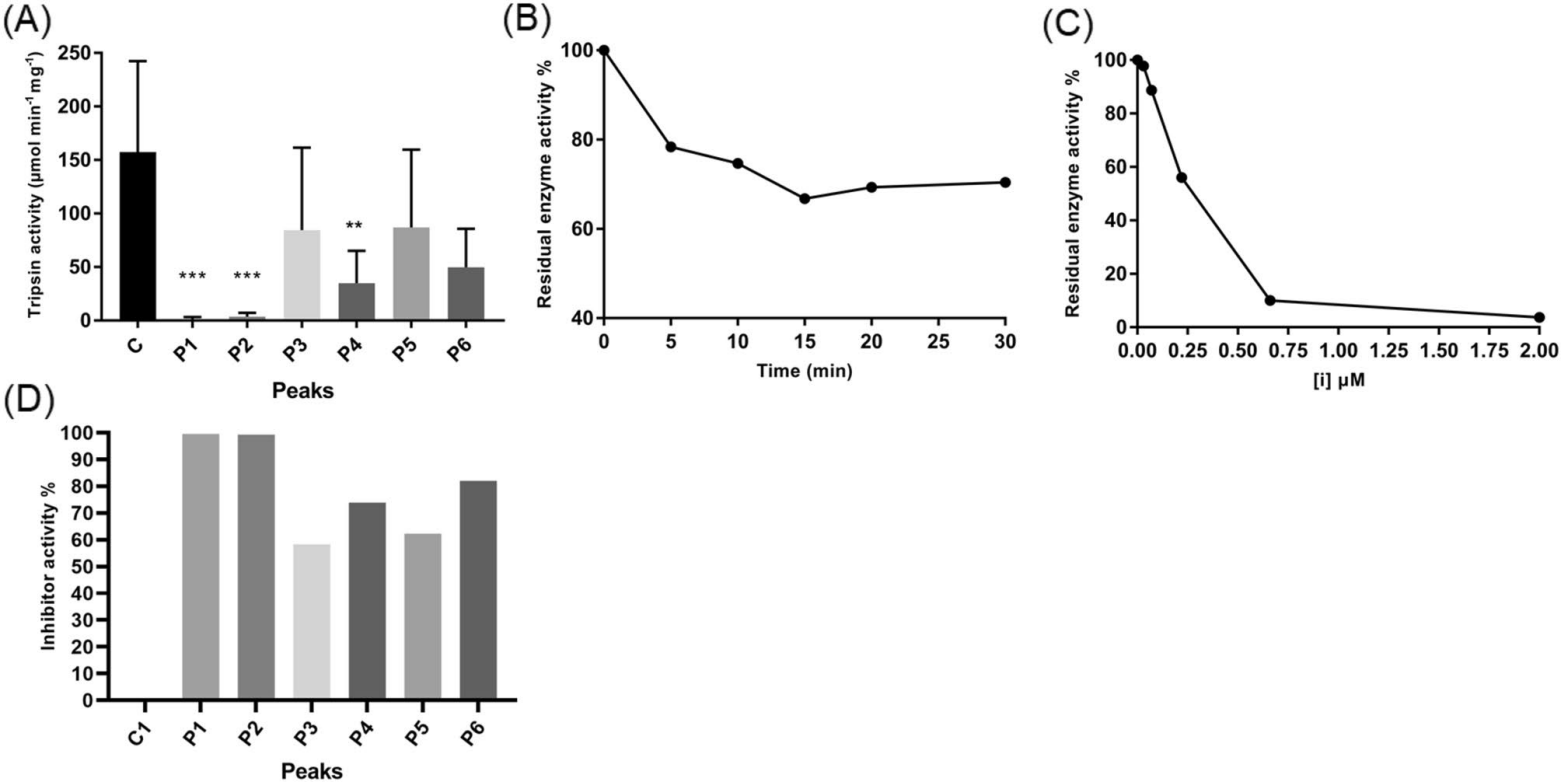
Inhibitory activity assays of NcTI and RP-HPLC purified inhibitory compounds. Inhibitory activity of peaks of the reverse phase chromatography profile as indicated in Fig. 1B. (**A**) Assay of inhibitory activity of peaks (P1–P6) of the chromatography profile (N = 3), C (control) of bovine trypsin without inhibitor compounds. **, ***Statistical analysis was performed using an unpaired Student T-Test. (**B**) Assay of time effect on inhibitory activity of P4 (NcTI). (**C**) Titration of inhibitor NcTI of *Nephilingis cruentata* in different concentrations (µM), was realized by the pre-incubation with trypsin (12 µg/µl) at 30 °C for 20 min. Trypsin residual activity was determined using ZFRMCA as substrate (N = 3). (**D**) Inhibitory activity of peaks (P1–P6) in percentage of inhibition (%), C (control) is 0% of inhibitory activity.

Anion exchange batch in the sequence of pH and thermal treatment and sedimentation resulted in a fraction with 100% trypsin inhibitory activity (fraction 2, Fig. 1A). Chromatographic separation of batch fraction 2 resulted in six main peaks (P1-P6) (Fig. 1B) with trypsin inhibitory activities in a range of 70 to 100% inhibition (Fig. 2A,D). Peaks named as 5a and 5b were pooled and named as P5. Figure 2B shows the effect of the time-dependent inhibitory activity of P4 inhibitor on bovine trypsin evidencing that P4 is a slow tight binding inhibitor, requiring at least 15 min of pre-incubation for trypsin inhibition. This inhibitor was named NcTI (Nc: Nephilingis cruentata, and TI: trypsin inhibitor). The titration of inhibitor NcTI (Fig. 2C) resulted in a K_D_ value of 30.25 nM ± 8.13.

**Figure 3:**
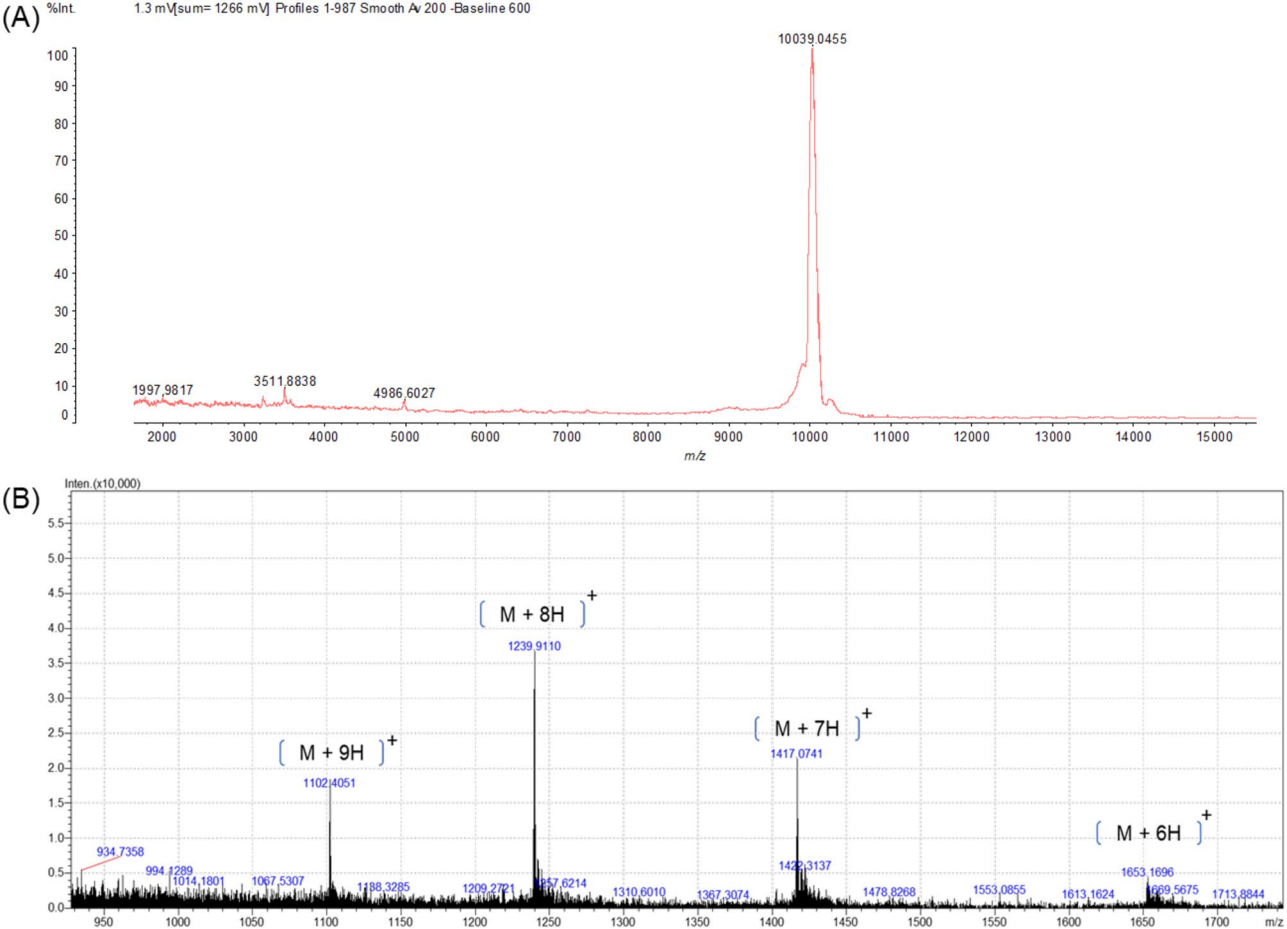
Mass spectrometry analysis of NcTI, Peak 4. (**A**) Mass spectrum of peak P4 obtained by MALDI-TOF, positive linear mode using sinapinic acid as a matrix, peak with a mass of 10,039.0455 Da. (**B**) Mass spectrometry profile obtained by LC–MS of P4, in positive mode. Ions of the ionic envelope of NcTI m/z = 1239.9110, 1102.4051, and 1417.0741 correspond to the average mass of 9912.3680 Da by deconvolution.

In Fig. 3A, MALDI-TOF/MS analysis evidenced that P4 (retention time of P4 = 24.40 min elute is in approximately 50% of solution B, in Fig. 1B) is an isolated molecule with a mass spectrum of the peak and their ion of greater relative intensity with a monoisotopic mass of 10,039.0455 Da (minus the mass 1 u of H^+^ or minus the mass 23 u of Na^+^). This data was corroborated by SDS-PAGE (Fig. 1C) where the protein profile of compounds purified from P4 shows only one protein band (Fig. 1C—lane 4). Relative migration (Rm = distance migrated by the protein/distance migrated by the dye front) of the band and molecular mass markers pattern as a standard curve resulted in a protein band of 28.78 kDa.

In the analysis performed using LC–MS (Fig. 3B), P4 presented mass spectra for the deconvolution calculation. The mass measured using the ion's envelope with values of m/z = 1239, 1102, 1417, and 1653 measured with different states of charge is 9912.3680 Da ± 0.75.

Proteomic analysis of the P4 samples using a database from *Nephilingis cruentata* (GenBank assembly project: GEWZ00000000.1; transcriptomic databases of *Nephilingis cruentata*) resulted in 38 unique peptides (Supplementary Table S1) having been detected by mass spectrometry de novo analysis. These peptides were identified in the sequence of the transcriptome contig 6834 from the database (Supplementary Fig. S1). Peptides identified provided 72% coverage of contig 6834. Contig 6834 is a transcript that has a high expression (2500 reads per kilo base of transcript per million reads mapped, RPKM) in the transcriptome of fastened animals^1^. These results prove the presence of NcTI in fasted animals. NcTI, as expected by the biochemical results (high thermal stability, high stability at low pH) is a protein rich in cysteine residues.

NcTI has a theoretical molecular mass of 11,842 Da and a theoretical pI of 5.36 (https://web.expasy.org/compute_pi). The isoelectric point of NcTI is characteristic of an acid protein^13^. The amino acid sequence was predicted as containing a signal peptide Sec/SPI (probability of 0.9994% according the signalP 6.0 software)^14^ with the molecular mass of the mature protein of 9912.38 Da. This result is in accordance with previous MALDI-TOF/MS and LC–MS analysis data.

### NcTI amino acid sequence characteristics

A search for amino acid sequences similar to NcTI was conducted in the MEROPS inhibitor database^15^, using the BLASTp algorithm. However, no significant result was found with the most similar sequences being the peptidase inhibitor from *Gallus gallus* from the family I8 (accession ID: MPEP0606623), with 29.5% of identity and an e-value of 0.34, and the *Ciona intestinalis* inhibitor peptidase of the I63 family (accession ID: MER0148068), with 45.2% identity and an e-value of 0.85.

**Figure 4:**
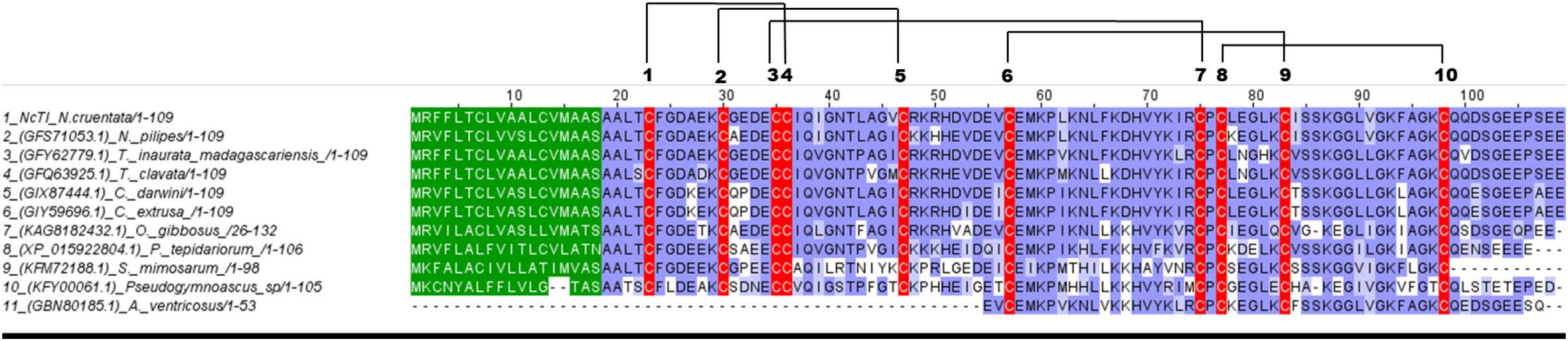
Alignment of NcTI and its best hits sequences from the NCBI database. Cys residue is in red, signal peptide in green. Blosum 62 score shows the conserved residues, and lines that link the residues Cys indicate disulfide bonds, according to the tridimensional prediction for NcTI. 100% 1-seq represents NcTI while the other sequence labels of the alignment represents the sequences from *Nephila pilipes* (2-seq, GFS71053.1); *Trichonephila inaurata madagascariensis* (3-seq, GFY62779.1); *T. clavata* (4-seq, GFQ63925.1); *Caerostris darwini* (5-seq, GIX87444.1); *C. extrusa* (6-seq, GIY59696.1); *Oedothorax gibbosus* (7-seq, KAG8182432.1); *Parasteatoda tepidariorum* (8-seq, XP_015922804.1); *Stegodyphus mimosarum* (9-seq, KFM72188.1); *Pseudogymnoascus sp*. (10 seq, KFY00061.1); and *Araneus ventricosus* (11-seq, GBN80185.1).

Ten putative homologous sequences were found in the complete NCBI protein database. These sequences were obtained from nine different spider and one fungi species, *Pseudogymnoascus* sp.^16^, using BLASTp. Figure 4 shows the alignment of NcTI amino acid sequence with these ten putative homologous sequences. These sequences have a minimum value of 50% identity and 80–100% protein coverage in comparison to NcTI. The alignment of the amino acid sequences shows a high conservation in almost all residues including the ten cysteines composing the five disulfide bonds of the protein.

**Figure 5:**
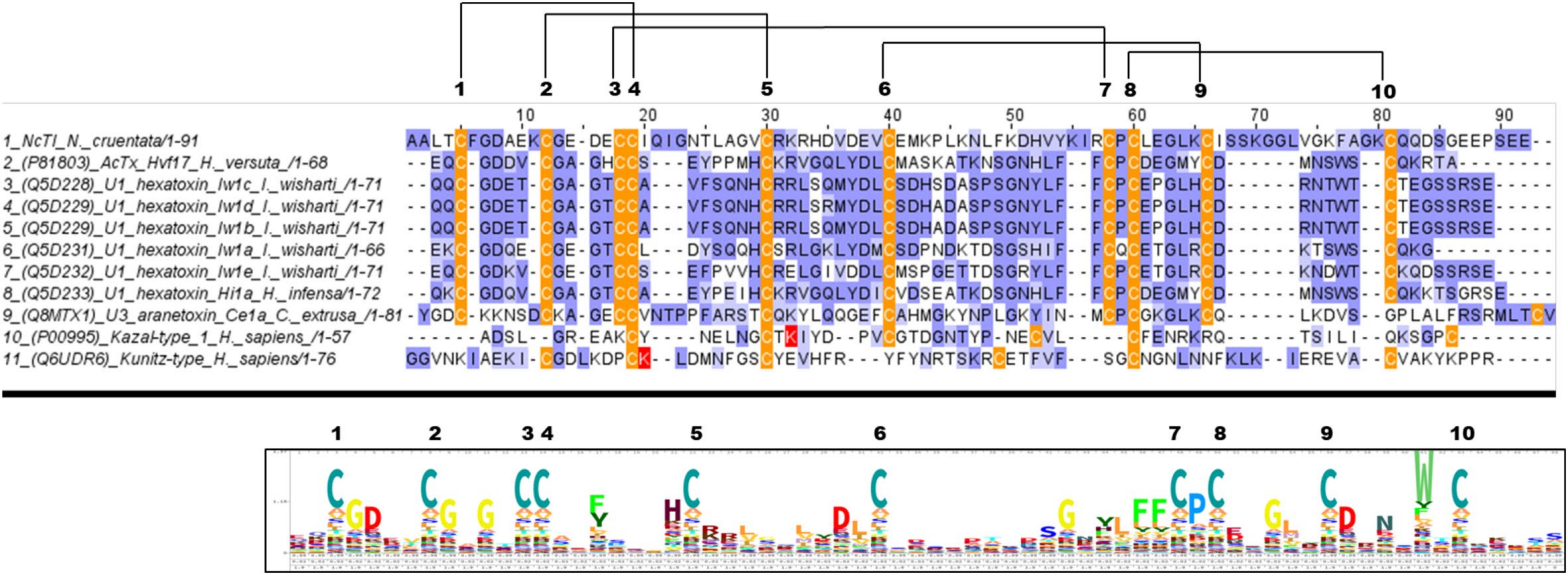
Amino acid sequence alignment of NcTI with ACTX-Hvf17, and representatives of the MIT-like atracotoxin family. Cys residues are shown in orange, and Lys residues, representing the reactive site residues of the inhibitors of the Kazal-type and Kunitz-type, are shown in red. Blosum 62 score shows the conserved positions, and lines connecting the Cys residues indicate disulfide bonds. Uniprot accession numbers and percentage of identity in relation to NcTI. 100% 1-seq: NcTI of *Nephilingis cruentata*; 20.59% ACTX-Hvf17 of *Hadronyche versuta* (2-seq, P81803); 26.76% U1 Hexatoxin-Iw1c of *Illawarra wisharti* (3-seq, Q5D228); 26.76% U1 Hexatoxin Iw1d of *I. wisharti* (4-seq, Q5D229); 26.76% U1 Hexatoxin Iw1b of *I. wisharti* (5-seq,Q5D230); 22.73% U1 Hexatoxin Iw1a of *I. wisharti* (6-seq,Q5D231); 32.39% Hexatoxin Iw1e of *I. wisharti* (7-seq, Q5D232); 20.83% U1 Hexatoxin Hi1a of *Hadronyche infensa* (8-seq, Q5D233); 23.38% U3 Aranetoxin-Ce1 of *Caerostris extrusa* (9-seq,Q8MTX1); 17.86% Serine protease inhibitor Kazal-type 1 (10-seq, P00995); 15.69% Kunitz-type protease inhibitor 4 (11-seq, Q6UDR6). The logo shows a graphic representation of the amino acid sequence conservation in MIT-like atracotoxin family.

An amino acid sequence alignment of NcTI and sequences from the MIT (mamba intestinal toxin)-like atracotoxin family (PF17556) (Fig. 5), shows several conserved regions among them (global identity value of 32%). Moreover, all sequences have ten cysteine residues, which are involved in the formation of the disulfide bonds C1–C4, C2–C5, C3–C7, C6–C9, and C8–C10. Representative sequences of inhibitors of the Kazal family (UniProt accession number: P00995, MEROPS Family I02) and Kunitz (UniProt accession number: Q6UDR6, MEROPS Family I03) were also aligned with peptides from the MIT-like atracotoxin family and with NcTI (Fig. 5), to analyze whether there would be some homology in the positions of the cysteine and reactive site residues, as the residues in these positions are used to classify these inhibitors^15^. The alignment did not show conservation in the cysteine and their correspondent disulfide bonds (C1–C5, C2–C4, and C3–C6 for Kazal-type inhibitor and C1–C6, C2–C4, and C3–C5 for Kunitz-type), and the identity between NcTI and these inhibitors converge to 17.86% and 15.64%, for the Kazal and Kunitz-type inhibitor sequences, respectively.

### NcTI predicted tridimensional structure

**Figure 6:**
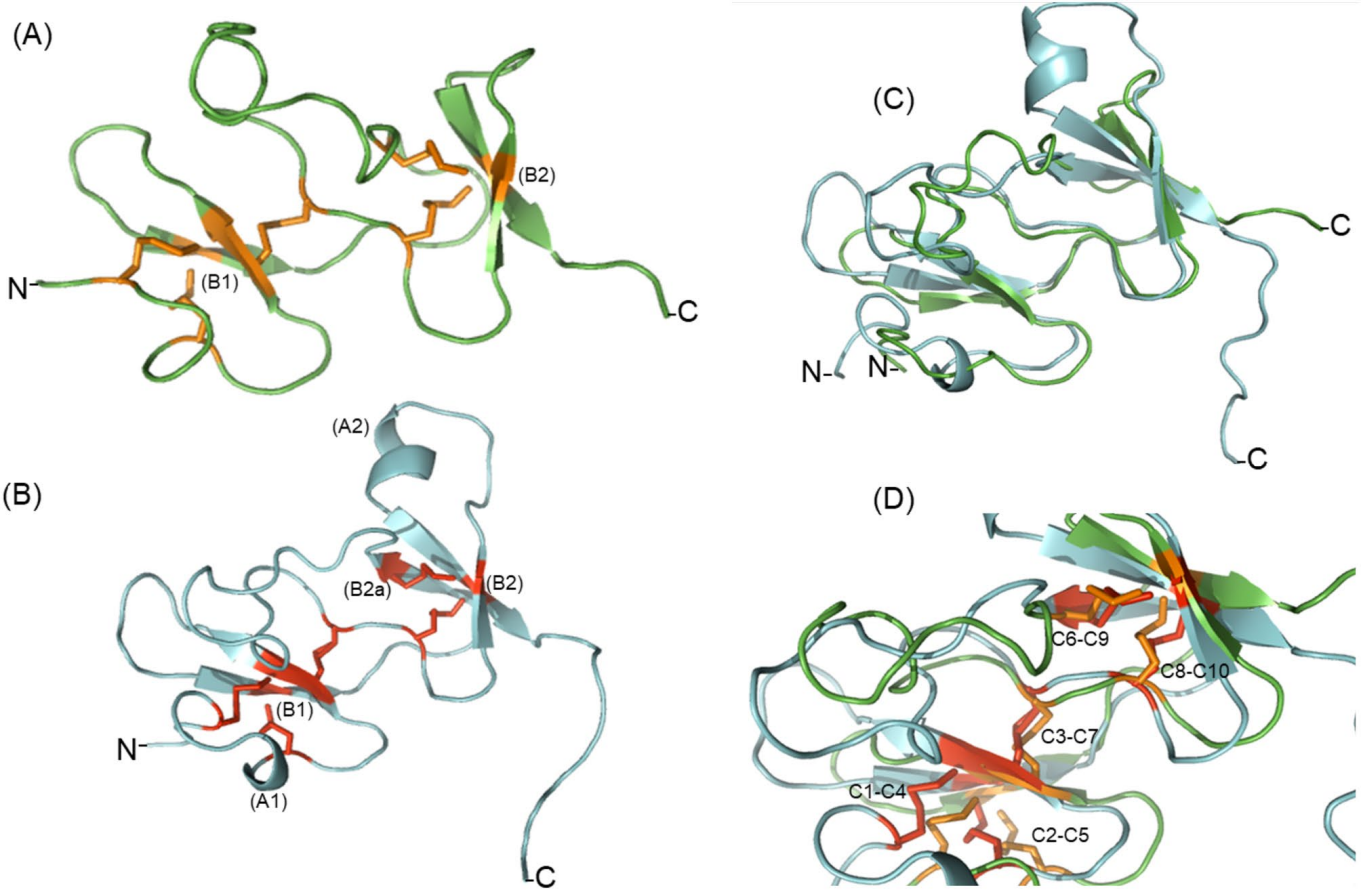
Comparison between the tridimensional structures predicted for NcTI and ACTX-Hvf17. (**A**) Tertiary structure predicted to ACTX-Hvf17. (**B**) Tertiary Structure predicted to NcTI. (**C**) Comparison of the structures of NcTI and ACTX-Hvf17. (**D**) Overlapping of disulfide bonds (C1–C4, C2–C5, C3–C7, C6–C9, and C8–C10) between the structures of NcTI and ACTX-Hvf17.

The predicted NcTI tridimensional structure has two antiparallel beta-sheet cores (B1 and B2), and two alpha helices (A1 and A2), besides the predominance of unstructured regions (Fig. 6B, Supplementary Fig. S2). A comparison with the ACTX-Hvf17 predicted structure (alpha fold accession number: P81803; https://alphafold.ebi.ac.uk), a spider representative of MIT-like atracotoxin family (Fig. 6A), showed a divergence in their secondary structure, as the ACTX-Hvf17 has no alpha helix predicted (Fig. 6C). However, based on the predicted structures, five disulfide bonds, identical to the pattern of cysteines of the MIT1-like atracotoxin family (C1–C4, C2–C5, C3–C7, C6–C9, C8–C10), are present in both proteins (Fig. 6D).

### Molecular docking analysis of the interaction between NcTI and bovine trypsin

**Figure 7:**
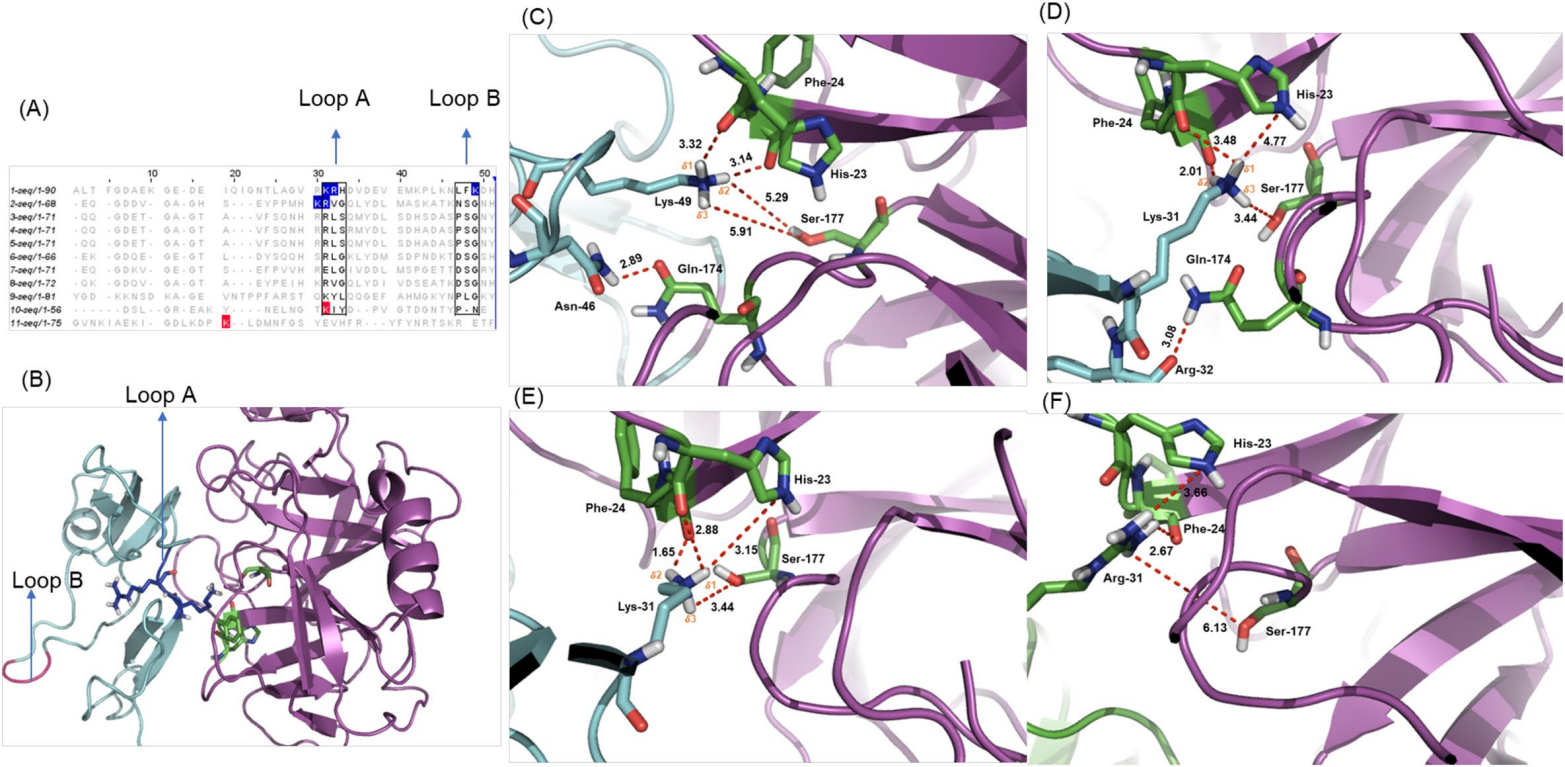
Molecular docking simulations between the inhibitor NcTI with the bovine trypsin (PDB:1S0Q). (**A**) Amino acid sequence alignment of NcTI with representative members of the MIT-like atracotoxin family. The residues in blue and the squares indicate the residues that are the possible candidates for the reactive site of the inhibitor. (**B**) Representation of the candidate loops to interact with the active site of trypsin with the NcTI structure colored in cyan, trypsin in purple and the trypsin residues His^23^, Phe^24^, and Ser^177^ are in green. (**C**) Molecular docking with NcTI-Lys^49^ as an active residue, showing the residue interaction with the trypsin residues Phe^24^, His^23^, and the catalytic Ser^177^. (**D**) Molecular docking with NcTI-Arg^32^ as an active residue. (**E**) Molecular docking with NcTI-Lys^31^ as an active residue. (**F**) Molecular docking with ACTX-Hvf17Arg^31^ as an active residue and its interaction with the trypsin Phe^24^, His^23^, and the catalytic Ser^177^.

The tridimensional structure of NcTI unveiled two candidate loops to interact with trypsin binding site. As trypsin cleaves peptide bonds at the C-terminal side of arginine or lysine residues, candidate loops were chosen as those containing Lys and/or Arg at their surface. The selected candidates were composed either by a Lys^31^ and an Arg^32^ residue (loop A) or only a Lys^49^ residue (loop B) (Fig. 7A, B). Similarly, the loop containing the residues Lys^30^ and Arg^31^ were selected as candidates for trypsin interaction in the ACTX-Hvf17 structure, based on the amino acid sequence alignment with NcTI (Fig. 7A). Molecular docking simulations between these putative inhibitors and bovine trypsin were then conducted using each one of these residues as active residues (residues directly involved in the interaction) in Haddock web server simulations^17,18^. The best model of interaction for each docking simulation according to Haddock (the best structure from the top cluster) is shown in the Fig. 7C–F and described in the Supplementary Table S2.

The molecular docking simulations using the Lys^49^ residue (loop B) as the only active residue on Haddock, shows that the side chain of Lys^49^ may interact with His^23^, Phe^24^, and Ser^177^ (corresponding to His 40, Phe 41, and Ser 195 bovine chymotrypsin numbering) in the trypsin structure, through hydrogen bonds, with distances of 3.14 Å with Phe^24^ (NHδ2^Lys^ and CO^Phe^), 3.32 Å with His^23^ (NHδ1^Lys^ and CO^His^), and 5.91–5.29 Å for interactions between the hydrogens of Lys^49^ and the oxygen of the catalytic Ser^177^ (NHδ2/δ3^Lys^ and OH^Ser^ hydrogens as numbered) (Fig. 7C).

Molecular docking simulations using only the loop A residue NcTI-Arg^32^ as an active residue shows that this particular residue may form a hydrogen bond only with the Gln^174^ residue of the bovine trypsin (Fig. 7D). This result shows that Lys^31^ is a better candidate to interact with trypsin, mimetizing their canonical substrate P1 residue.

In molecular docking simulations with the loop A residue NcTI-Lys^31^ as the only active residue, the best model of interaction predicted (Fig. 7E) reveals the possibility of interaction with the trypsin specificity/binding residues His^23^, Phe^24^, and the catalytic Ser^177^ (corresponding to Ser^195^- bovine chymotrypsinogen), with smaller distances between atoms, compared to previous simulations. The lysine side chain would form hydrogen bonds at distances of 2.88 Å or 3.15 Å with His^23^ (NHδ1^Lys^ and CO^His^ or NH^His^) 1.65 Å with Phe^24^ (NHδ2^Lys^ and CO^Phe^) and 3.44 Å with catalytic Ser^177^ (NHδ3^Lys^ and COH^Ser^).

For ACTX-Hvf17, the molecular docking simulations using the Arg^31^ residue as the active residue showed a better result than with Lys^30^. In the simulations with Arg^31^, this residue interacted with the residues His^23^, Phe^24^, and Ser^177^ of trypsin (Fig. 7F), whereas the side chain of Lys^30^ was distant from the active site of the enzyme. The predicted mode of interaction between ACTX-Hvf and the bovine trypsin shows a higher distance on the hydrogen bond between the Arg^31^ and the catalytic Ser^177^ (6.13 Å) than that between NcTI-Lys^31^ and Ser^177^ (3.44 Å), which may represent a weaker interaction with this enzyme.

## Discussion

The enhancement of high throughput data on spiders´ digestion and digestive system indicate the presence of molecules on both, the digestive fluid and MMD, similar to toxin/venom proteins previously described as venom exclusive molecules^1,2^. As in venom, the digestive fluid and digestive tract also has cysteine-rich molecules, forming distinct structures and playing diverse roles. On venom secretion, these cysteine-rich molecules are usually very effective toxins acting on the prey ion channel being responsible for its paralysis^6^. Besides the high content of cysteine residues and disulfide bonds, these proteins show very low conservation on their amino acid sequences, which confers to them different functions and specificities^7^. This statement seems to be true for venom cysteine-rich peptides but there is little information about those peptides from digestive secretions.

Due to that, the isolation and characterization of molecules with potential inhibitory activities derived from the spiders’ digestive system is necessary. Since several of these molecules are rich in disulfide bonds, the acidification and thermal pre-treatment during the preparation steps help to enrich the samples with these molecules, since most of the proteins are denatured under these conditions, facilitating further purification steps^19^. Proteins previously identified by proteomic analysis of the whole midgut from *Nephilingis cruentata* pointed to candidate peptidase inhibitor molecules with acidic pI (in the range of 5.4), which favors the use of anion exchange resins for isolation^20^. In our work, the *N. cruentata* MMD sample prepared using pH and thermal treatment showed high inhibitory activity on bovine pancreatic trypsin (Supplementary Fig. S3). Electrophoresis and MALDI-TOF/MS analysis of this purified sample evidenced an isolated protein, which was further identified by proteomic sequencing as the protein coded by the transcript contig 6834, previously described for the digestive system of the spider *N. cruentata*^1^. Inhibitor molecular mass prediction from the NcTI mRNA sequence indicates a protein with a molecular mass of 11,842 Da in the presence of the signal peptide. The data of mass spectrometry reinforces the indication of a signal peptide, since the masses obtained through the MALDI-TOF/MS techniques, and mainly by LC–MS, are approximate to the mass theoretical prediction without the presence of the signal peptide. Considering the different measurements of the spectrum, we suggest that the mass of this mature protein is 9912.3680 Da. There was some contradiction concerning the mass of NcTI determined by SDS-PAGE, which shows an approximate mass value of 28.78 kDa and mass spectrometry. NcTI has characteristics of proteins that migrate abnormally when subjected to electrophoretic migration, such as the Gir2 protein from *Saccharomyces cerevisiae*. NcTI is rich in acidic amino acid residues, which acquires a high charge negative at pH 7 resulting less binding at the SDS, reducing migration in the gel, and an N-terminal PEST sequence (KCQQDSGEEPSEE) with a score of 13.56 based on the PEST algorithm^21,22^. These motifs are found in proteins that are rapidly degraded, in addition, NcTI shows high stability to temperature, pH variation, and unstructured regions. All the mentioned characteristics are also found in Gir2, therefore, we consider that NcTI may also present an anomalous migration under the conditions of the SDS-PAGE technique.

Sequences similar to NcTI were also identified in other species of spiders with genome sequences available at the NCBI database. However, the proteins coded by these sequences were not isolated and have their biological functions still unknown^23–25^. The signal peptides of these putative proteins have a conserved consensus sequence MRXFLTCLVASLCVMAASA (Fig. 4). The only exception was the sequence of *A. ventricosus* (GBN80185.1 - 11-seq), which is fragmented at the N-terminal region and do not have a predicted signal peptide at this region as well as the residues between the positions 45 to 79, a very conserved region.

The sequences identified in the other spiders, as well as NcTI, are putative homologs of ACTX-Hvf17, which belongs to the MIT-like atracotoxin family (2-seq in Fig. 5). Members of this protein family were described in the venom of three species of spiders, the Australian funnel-web, *Illawarra wisharti*, *Hadronyche versuta*, and *Hadronyche infensa*, for which the components of the venom exhibited excitatory neurotoxin properties^26^. However, the biological function of the MIT-like atracotoxin family has not yet been described. Structurally, atracotoxins resemble the structure of colipases. However, although the conserved structure, studies have shown that peptides of the atracotoxin family are not able to act as colipases, nor increasing the contraction of smooth muscle in rats, or interacting with prokineticin receptors that are targets of the mammalian AVIT family^26,27^.

Biochemical assay of NcTI protein isolated pointed towards an inhibitor of trypsin. Time-dependence assay (Fig. 2B) results indicate that NcTI is a time-dependent inhibitor. This behavior is typical of tight binding competitive reversible inhibitors. Reversible inhibitors in general follow Michaelis–Menten kinetics, in that the concentration of enzyme [E] is less than Ki and the concentration of complex [EI] is much lower compared to the concentration of free inhibitor [I]^28^. However, characteristics of strong binding, high affinity, and fast association with the target enzyme, whose dissociation from the [EI] complex is extremely slow, characterize tight-binding inhibitors^29^. For this reason, these properties do not allow the use of equilibrium assumptions, and the use of steady-state approximations, in this situation the calculation of Ki, which measures the association constant of the enzyme and inhibitor, is compromised. Morris shows that classic double reciprocal plots are not suitable for tight-binding inhibitors^29,30^. In this case, the use of methodologies to calculate IC_50_ and K_D_ values is more appropriate for tight-binding inhibitors^31^.

The kinetic assays (Fig. 2B) show that NcTI has the characteristic of time-dependent for binding with bovine trypsin, because many of the tight-binding inhibitors have a slow onset of inhibition, making a pre-incubation necessary to evaluate the inhibition activity. Figure 2C shows that the association of [E] and [I], decreased initial trypsin concentration (measured by the percentage of residual specific activity) as the concentrations of NcTI increased, shifting the equilibrium towards the formation of the complex [EI] and reducing the initial concentration [I], that is another property recognized as specific for tight-binding inhibitors, with a 1:1 binding characteristic, almost stoichiometric, these inhibitors are useful to titrate enzymes^28,32^.

The experimental data show that NcTI is a reversible tight-binding trypsin inhibitor, with a K_D_ value of 30.25 nM ± 8.13 nM. This value when compared with other ​​serine peptidase inhibitors from spiders' venom, such as HWTX-XI with K_D_ value of 0.23 nM against trypsin, shows that NcTI has 131 times less inhibitory capacity than HWTX-XI. Compared with the serine protease Inhibitor recombinant AvKTI (Kunitz type), with the value of Ki 7.34 nM, NcTI has 4.1 times less inhibitory capacity^9,10^. The variation of K_D_ values between HWTX-XI, AvKTI and NcTI is possibly due to structural differences between these molecules since NcTI is a larger protein that has more structured regions, leading to different number of sites of interaction with trypsin. SBTI (soybean trypsin inhibitor), the standard trypsin inhibitor, has a molecular mass similar to NcTI but has a K_D_ value of 500 nM^33^, therefore NcTI is more effective in comparison to SBTI.

Although, the inhibitor NcTI shares the structural MIT1-like motif, the maintenance of this structural motif also occurs in other organisms, such as the AVIT protein family in mammals, in the *Bombina variegata* frog species, and *Dendroaspis polylepis* that has intestinal toxin 1^34–36^. The structural analysis of NcTI indicates that this molecule shows a more complex tertiary structure than ACTX-HVf17 and may interact with higher affinity to the bovine trypsin.

The NcTILys^31^ residue can be considered the reactive site of these proteins as docking simulations resulted in models in which the side chain of Lys^31^ forms hydrogen bonds between with three trypsin residues. The side chain of Lys^31^ interacts with two residues of the active site of trypsin, the catalytic Ser^177^ (position 195 in bovine chymotrypsin numbering) and His^23^ (position 40 in bovine chymotrypsin numbering). In general, the catalysis mechanism of serine peptidases uses Ser^195^ to perform a nucleophilic attack on the carbonyl group of the peptide bond. The result is the cleavage of the peptide bond at the terminal carboxyl region of the target proteins^37,38^.

The vast majority of serine peptidase inhibitors, such as the Kazal, Kunitz, and Bowman-Birk family, have a wide variety of structures and sequences. They work through the standard Laskowski mechanism, inserting a loop with a reactive site at the catalytic site of the target enzyme. The reactive site of these inhibitors is configured to be similar to the substrate, interacting with both enzyme subsites S1, S2, S1´ and S2´ of the active site of serine peptidases^39,40^. The main hypothesis established is that the inhibitor NcTI appears to act in the same way, inserting the loop A into the active site of bovine trypsin and interacting by hydrogen bonds between the side chain of Lys^31^ with Ser^177^, then the formed scissile bond follows the standard mechanism of Laskowski-type inhibitors without undergoing complete hydrolysis of the reactive site^41^. Based on these molecular docking results, experiments based on crystallographic or NMR determination of the enzyme/inhibitor complex are promising. Thus, the next steps of this work will be the cloning, expression, structural resolution and biological assays for functional analysis and application.

## Methods

### Extraction of diverticula from the middle intestine MD and sample preparation

Adult females of *Nephilingis cruentata* (Araneae: Nephilidae) collected around the Butantan Institute were kept in the laboratory under natural humidity and temperature conditions. After two weeks of fasting, the spiders were immobilized in a carbon dioxide chamber for 10 min. Then, the animals were dorsally allocated on a paraffin plate. The midgut and midgut diverticula (MD) at the opisthosoma region were isolated in 0.3 M NaCl solution and stored at − 20 °C until use.

A total of 8 MDs per biological replicate (biological triplicate) were isolated and homogenized in 3 ml of 0.1 M sodium acetate buffer pH 3.5 with the aid of a Potter–Elvehjem homogenizer. Homogenate samples were centrifuged for 30 min at 16,000 RCF at 4 °C. The soluble fraction was separated and boiled at 100 °C for 5 min and centrifuged sequentially for 30 min at 16,000 RCF at 4 °C, the soluble samples were stored at -20 °C until use.

### Bovine trypsin and inhibition activity assays. Protein fluorescence estimation

The inhibition activity was measured against bovine pancreatic trypsin (Sigma: 6502), and the catalytic activity was determined using stock solutions substrate of 1.0 mM carbobenzoxy-Phe-Arg-7-amido-4-methyl-coumarin (ZFRMCA Sigma: C9521) 1.0 mM dissolved in dimethyl sulfoxide. Stock solutions were diluted 1:9 in 0.1 M Tris–HCl buffer pH 8.0 to a final concentration of 0.1 mM, as substrate. Pre-incubation enzyme assays were performed at a mixture of 1:1 (v/v) ratio of trypsin (solution of trypsin final concentration 12 ng/µl) and inhibitor source (solution of trypsin final concentration 12 ng/µl for µg of inhibitor) for the peaks purified by RP-HPLC, and incubated at 30 °C for 20 min. Substrate was added to preincubated mixtures and submitted to incubation at 30 °C in a Gemini Spectrofluorimeter (Molecular Devices). The kinetics readings were performed at 10 min intervals for 1 h. The product was measured by Gemini Spectrofluorimeter (Molecular Devices) with 340 nm of excitation and 440 nm of emission. Positive control (with no inhibitor) was set as 100% bovine trypsin activity. For assaying the time effect on inhibitory activity, pre-incubation was done at different times of inhibitor NcTI at 4 µM with trypsin bovine at 12 ng/µl at 30 °C and residual activity was evaluated. Statistical analysis of those data was performed using an unpaired Student T-Test. Protein determination of the samples was performed by Qubit (Thermo Fisher Scientific Qubit Protein Assay Kit).

### Isolation of serine peptidase inhibitors by anion exchange batch

QAE-Sephadex (sigma: Q25120, 0.5 g) was resuspended in 12.5 ml of 25 mM ammonium bicarbonate (NH_4_HCO_3_) at pH 8.5 and incubated for 18 h at 25 °C, under agitation. After incubation, it was centrifuged for 5 min at 500 RCF, discarding the supernatant, and the process was repeated twice. Sample supernatants described at item 4.1 were added to 8 ml of 25 mM ammonium bicarbonate buffer at pH 8.5 and mixed and incubated for 3 h to previously equilibrated QAE resin. The elution process was carried out with three solutions (1): 12.5 ml of 25 mM ammonium bicarbonate pH 8.5. Solution (2): 12.5 ml of 25 mM ammonium bicarbonate with 1 M of NaCl pH 8.5 and solution (3): 12.5 ml of 25 mM ammonium bicarbonate pH 3.5. For each elution step, centrifugation was performed at 500 RCF for 30 min at 25 °C, the supernatant was removed, concentrated by vacuum centrifugation to a final volume of 3 ml. The eluted samples were named (1), (2), and (3), referring to the solutions used and individually stored at -20 °C. The inhibitory activity of each fraction was evaluated using the methodology exemplified in item 4.7.

### Purification of serine peptidase inhibitors by RP-HPLC

The fractions with inhibitory activity from the batch isolation were purified by reversed-phase in a binary HPLC system (20A Prominence, Shimadzu Co., Japan) at a concentration of 1 mg ml^−1^. The sample was loaded in a C18 column (5 μm; 100 Å; 250 × 4.6 mm), with a flow rate of 1 mL min^−1^ at temperature 40 °C. Peptide detection was performed at 214 nm. The mobile phase consisted of solution (A): water: TFA (999:1) and solution (B): water: ACN: TFA (900: 99: 1). A linear gradient in a range of 5% to 90% of solution (B) in 35 min, after 5 min isocratic elution with 5% the solution (B) was used for protein elution. The peaks were collected by time retention, concentrated by vacuum centrifugation, resuspended in ultrapure water, and evaluated for inhibitory capacity against bovine trypsin.

### SDS–Polyacrylamide gel electrophoresis

The samples eluted from chromatography on RP-HPLC (10 µg of protein) were loaded on 4–20% gradient SDS- PAGE gel kit Biocompare (www.biocompare.com) in reducing conditions. The samples were resuspended in sample buffer containing (60 mM) Tris–HCl buffer (pH 6.8), 2.5% SDS, (0.36 mM) β-mercaptoethanol, 10% (v/v) glycerol and 0.005% bromophenol blue (w/v) at 100 °C. The gel was electrophoresed at a constant voltage of 160 V for 1 h and stained with Coomassie brilliant blue G-250^42^.

### Matrix-assisted laser desorption ionization

Analyzes were performed using MALDI-TOF–MS (AXIMA Performance, Shimadzu Co., Japan) with peak purified by RP-HPLC added to 20 mg. ml^−1^ sinapic acid saturated in 50% acetonitrile and 0.1% trifluoroacetic acid as a matrix, and analyzes were performed in positive linear mode.

### Mass spectrometry

Liquid chromatography coupled to mass spectrometry analysis was used and performed in a mass spectrometer ESI-IT-TOF (20A Prominence, Shimadzu Co., Japan). Samples (RP-HPLC sample, 3 ug) were diluted in 0.1% acid formic and loaded in the spectrometer at a flow of 0.2 mL min^−1^ at 40 °C, and an isocratic profile of 50% acetronile solvent (B) with 50% water acid at 0.1% acid formic as solvent (A), spectra were collected in the range of m/z: 200 to 1950^43^. The raw data (mzxml) was uploaded to Peaks Studio 8.5 (Bioinformatics Solution Inc., Waterloo, Canada) and processed with new peptide sequencing and Peaks DB. The MS/MS spectra were searched in a custom database GEWZ00000000.1 compiled from the UniProt database.

### Titration of the inhibitor and apparent dissociation constant of the enzyme-inhibitor complex

Distinct samples of NcTI purified inhibitor dilutions (different dilutions from 4 µM at 0.25 µM) were assayed in the presence of bovine trypsin 12 ng/µL. The rectilinear portion of the plot of trypsin activity, measured against inhibitor concentration, extrapolates to a concentration of NcTI which is equivalent to the total concentration of trypsin. The activity of trypsin in the equivalence point (point in the titration curve above the intercept in the NcTI concentration axis) corresponds to the concentration of free enzyme, which is identical to that of free NcTI. Knowing the total concentration of enzyme, the concentration of free enzyme (*E*) and of free inhibitor (*I*), it is possible to calculate the concentration of trypsin-NcTI complex (*EI*). The apparent dissociation constant of trypsin- NcTI complex (*KD*) may be calculated by the equation (see details)^31,44^:

$$KD = \, \left[ E \right]\left[ I \right]/\left[ {EI} \right].$$

### Analysis by shotgun proteomics bottom-up

Proteomic analysis was performed with a gel slice of approximately 1 mm^2^, and the pieces were destained with 200 µL of decolorized solution (50 mM of NH_4_HCO_3_ in 25% ACN). In the next step, 200 µl ACN were added until the gel pieces got white, and in sequence ACN was removed by vacuum centrifugation (Speed Vac) for 10 min. After that, 100 mM dithiothreitol (DTT) was added (v/v) for 30 min at room temperature. After incubation DTT solution was discarded. The addition of ACN and vacuum centrifugation was repeated. The sample was then alkylated with 200 mM (v/v) iodoacetamide (IAA) at 25 °C for 30 min in the dark. After this procedure, the IAA solution was discarded, and the addition of ACN was repeated once more. The pieces of the gel after precedent were incubated for 18 h with 100 ng of bovine pancreatic trypsin (Sigma 6502), and the reaction was stopped with 10 µl of 10% acetic acid. The samples were concentrated by vacuum centrifugation and resuspended in 0.1% acetic acid solution. Aliquots of the grouped samples (n = 4) were analyzed in a Dionex Ultimate 3000 RSLCnano (Thermo Fisher Scientific) system connected to an Impact II mass spectrometer (Bruker Daltonics). The mobile phases used were as follows: Solution A: Water: Ac. Formic (999: 1) and solution B: ACN: Ac. Formic (999:1). The samples were injected and uploaded online into a Nano-trap Acclaim PepMap column (Dionex-C18, 100 Å, 75 μm × 2 cm) using 98% solution A for 2 min at a flow rate of 5 μL min^−1^ and then the peptides were eluted with a gradient of 5 to 40% solution B over 120 min at a flow rate of 350 nL min^−1^. Mass spectra were acquired in positive ion mode with MS precursors and MS/MS products were analyzed at 2 Hz in the mass range of 50–2000 m/z. Ramp-induced dissociation energy (ICD) parameters ranged from 7 to 70 eV.

### Sequence alignment analysis and molecular modeling

Alignments based on sequence similarity and conservation of cysteines pattern were used to determine the inhibitor's classification. The alignment of the sequence was generated by multiple sequence alignment using clustalW. Sequence analysis and visualization was performed in the Jalview software. Signal Peptide prediction was performed using SignalP - 6.0^14^. The NcTI and ACTX-Hvf17 tertiary structure were predicted using ColabFold: AlphaFold2 using MMseqs2. Image analyses were done at Pymol. The analyzes performed with the alpha fold tools demonstrate the quality of the models and estimate the reliability of the results^45^.

### Molecular docking

Trypsin and soybean trypsin inhibitor complex atomic coordinates (PDB ID: 1AVX) and bovine trypsin complexed with benzamidine atomic coordinators (PDB: 1S0Q) were retrieved from the Protein Data Bank (PDB). PIC: Protein Interactions Calculator software^46^ was used to identify protein–protein interactions between 1AVX peptide chains (A; trypsin and B; Trypsin inhibitor from *Glycine max*). Residues involved in protein–protein interactions such as hydrophobic interaction, main chain-main chain hydrogen bonds, side chain-side chain hydrogen bonds and main chain-side chain interactions were identified. Residues involved at 1S0Q and benzamidine interactions were also identified. Residues involved in S1, S2, S1´, S2´binding sites and catalytic residues (189, 193, 195, 190, 219, 220; 215; 195; 40) were identified and used to predict the contact P1 residues at NcTI. It was possible to choose the residues that were most likely to form hydrogen bonds between NcTI inhibitor residues and bovine trypsin.

Tridimensional structures of NcTI were predicted using AlphaFold 2, as mentioned previously. The structures were then submitted to HADDOCK docking prediction^17,18^ using standard parameters. The best models were resubmitted to analysis of interaction in PIC and visualized in Pymol software.

## Supplementary Information

Supplementary Information.

## Data Availability

The datasets generated and/or analyzed during the current study are available in: Proteomic data: the PRIDE repository, Data are available via ProteomeXchange (http://www.proteomexchange.org) with Project accession: PXD037594, Project https://doi.org/10.6019/PXD037594.
