# Supplementary material for: Spiders’ digestive system as a source of trypsin inhibitors: functional activity of a member of atracotoxin structural family
Source: Sci Rep. 2023 Feb 10;13:2389. doi: 10.1038/s41598-023-29576-y (PMC9918498; doi:10.1038/s41598-023-29576-y)
Supplement: Supplementary file 1 — Supplementary Information. [file 41598_2023_29576_MOESM1_ESM.docx]

**Supplementary material** **
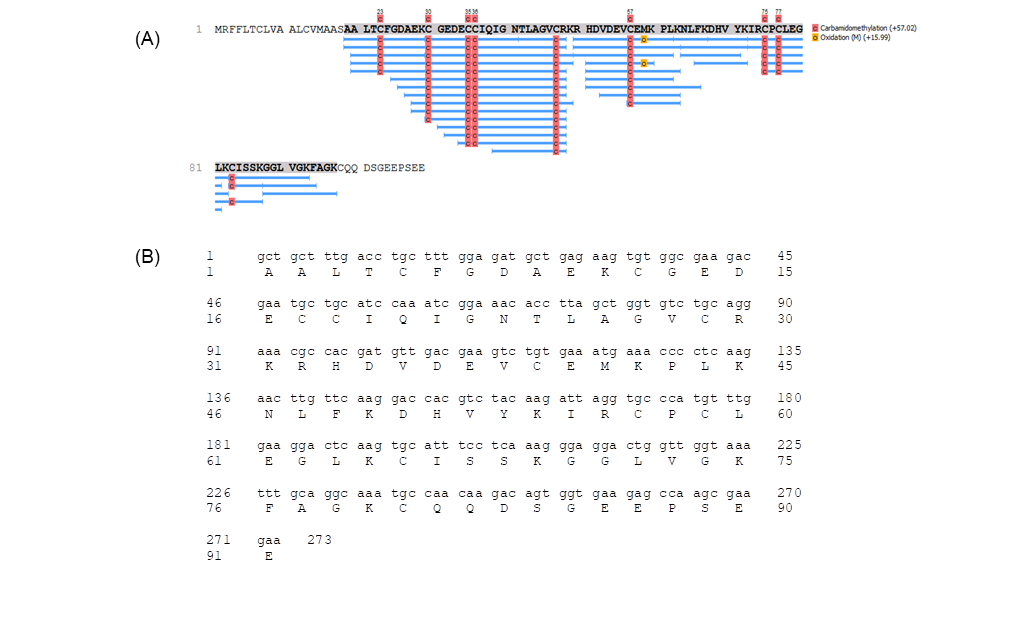
**

**Supplementary Fig. S1**. **Proteomic analysis and nucleotide and aminoacid sequence of NcTI**. (A) Coverage of inhibitor Nc6834, highlights for carbamidomethylation in red of residues Cys and yellow oxidation of residue of Met. As expected, the signal peptide region (N-terminal) was not detected. (B)  ORF  representation of nucleotide and amino acid sequence.

| Peak | Fasting transcriptome | -10lgP | Coverage (%) | Peptides | Unique | PTM | Avg. Mass | FDR |  |
| --- | --- | --- | --- | --- | --- | --- | --- | --- | --- |
| 4 | **Contig 6834** | **469.66** | **72** | **40** | **38** | **Y** | **11842** | **≥49** |  |
| Peptides | **Uniq** | **-10lgP** | **Mass** | **ppm** | **m/z** | **z** | **RT** | **Scan** | **PTM** |
| K.C(+57.02)GEDEC(+57.02)C(+57.02)IQIGNTLAGVC(+57.02)R.K | Y | 162.48 | 2210.9177 | 4 | 737.9828 | 3 | 76.88 | 24782 | Carbamidomethylation |
| A.ALTC(+57.02)FGDAEKC(+57.02)GEDEC(+57.02)C(+57.02)IQIGNTLAGVC(+57.02)R.K | Y | 162.43 | 3303.4087 | 5.6 | 1102.1497 | 3 | 79.94 | 26169 | Carbamidomethylation |
| D.AEKC(+57.02)GEDEC(+57.02)C(+57.02)IQIGNTLAGVC(+57.02)R.K | Y | 132.83 | 2539.0923 | 5.8 | 847.3763 | 3 | 71.53 | 22579 | Carbamidomethylation |
| K.C(+57.02)GEDEC(+57.02)C(+57.02)IQIGNTLAGVC(+57.02)RK.R | Y | 125.04 | 2339.0127 | 5.4 | 780.6824 | 3 | 71.6 | 22599 | Carbamidomethylation |
| A.ALTC(+57.02)FGDAEKC(+57.02)GEDEC(+57.02)C(+57.02)IQIGNTLAGVC(+57.02)RK.R | Y | 114.04 | 3431.5037 | 3.5 | 858.8862 | 4 | 77.25 | 24971 | Carbamidomethylation |
| R.HDVDEVC(+57.02)EMKPLK.N | Y | 113.14 | 1598.7433 | 3.7 | 400.6946 | 4 | 55.99 | 16634 | Carbamidomethylation |
| C.GEDEC(+57.02)C(+57.02)IQIGNTLAGVC(+57.02)R.K | Y | 93.71 | 2050.887 | 6.5 | 684.6407 | 3 | 76.48 | 24606 | Carbamidomethylation |
| K.RHDVDEVC(+57.02)EMKPLK.N | Y | 90 | 1754.8444 | 3 | 439.7197 | 4 | 55.33 | 16382 | Carbamidomethylation |
| F.GDAEKC(+57.02)GEDEC(+57.02)C(+57.02)IQIGNTLAGVC(+57.02)R.K | Y | 87.49 | 2711.1406 | 5.6 | 904.7259 | 3 | 72.85 | 23086 | Carbamidomethylation |
| D.EC(+57.02)C(+57.02)IQIGNTLAGVC(+57.02)R.K | Y | 74.6 | 1749.796 | 4.5 | 875.9092 | 2 | 74.63 | 23820 | Carbamidomethylation |
| G.EDEC(+57.02)C(+57.02)IQIGNTLAGVC(+57.02)R.K | Y | 72.11 | 1993.8656 | 6.1 | 997.9462 | 2 | 76.75 | 24725 | Carbamidomethylation |
| K.IRC(+57.02)PC(+57.02)LEGLK.C | Y | 71.31 | 1244.637 | 2.7 | 623.3275 | 2 | 60.44 | 18293 | Carbamidomethylation |
| R.HDVDEVC(+57.02)EMKPL.K | Y | 71.26 | 1470.6483 | 6.4 | 491.2265 | 3 | 64.68 | 19932 | Carbamidomethylation |
| S.AALTC(+57.02)FGDAEKC(+57.02)GEDEC(+57.02)C(+57.02)IQIGNTLAGVC(+57.02)R.K | Y | 69.71 | 3374.4458 | 6.6 | 844.6243 | 4 | 80.11 | 26248 | Carbamidomethylation |
| R.C(+57.02)PC(+57.02)LEGLK.C | Y | 68.98 | 975.4518 | 0.5 | 488.7334 | 2 | 50.59 | 14786 | Carbamidomethylation |
| A.EKC (+57.02) GEDEC (+57.02) C(+57.02) IQIGNTLAGVC(+57.02)R.K | Y | 67.34 | 2468.0552 | 8.7 | 823.6995 | 3 | 72.04 | 22749 | Carbamidomethylation |
| K.RHDVDEVC(+57.02)EMKPL.K | Y | 60.11 | 1626.7494 | 5.7 | 407.697 | 4 | 62.31 | 19008 | Carbamidomethylation |
| G.DAEKC(+57.02)GEDEC(+57.02)C(+57.02)IQIGNTLAGVC(+57.02)R.K | Y | 59.18 | 2654.1194 | 4.1 | 885.7173 | 3 | 74.51 | 23779 | Carbamidomethylation |
| A.EKC(+57.02)GEDEC(+57.02)C(+57.02)IQIGNTLAGVC(+57.02)RK.R | Y | 57.57 | 2596.1501 | 8.1 | 650.05 | 4 | 67.46 | 20989 | Carbamidomethylation |
| A.ALTC(+57.02)FGDAEK.C | Y | 52.93 | 1110.5016 | 2.3 | 556.2593 | 2 | 52.06 | 15271 | Carbamidomethylation |
| R.HDVDEVC(+57.02)EMKPLKNLF.K | Y | 50.34 | 1972.9386 | 2.7 | 494.2433 | 4 | 78.03 | 25301 | Carbamidomethylation |
| K.C(+57.02)ISSKGGLVGK.F | Y | 47.57 | 1104.5961 | 2.5 | 553.3067 | 2 | 24.99 | 6842 | Carbamidomethylation |
| S.AALTC(+57.02)FGDAEK.C | Y | 45.84 | 1181.5387 | 2.9 | 591.7783 | 2 | 54.38 | 16067 | Carbamidomethylation |
| K.DHVYK.I | Y | 44.27 | 660.3231 | 6.6 | 331.171 | 2 | 51.45 | 15071 |  |
| R.C(+57.02)PC(+57.02)LEGLKC(+57.02)ISSK.G | Y | 44.1 | 1550.7255 | 6 | 517.9189 | 3 | 63.41 | 19410 | Carbamidomethylation |
| K.NLFKDHVY.K | Y | 40.24 | 1034.5186 | 5.3 | 518.2693 | 2 | 62.56 | 19114 |  |
| D.VDEVC(+57.02)EMKPLK.N | Y | 38.16 | 1346.6573 | 2.9 | 449.8944 | 3 | 52.09 | 15287 | Carbamidomethylation |
| L.FKDHVYK.I | Y | 38.05 | 935.4865 | 3.3 | 312.8371 | 3 | 52.52 | 15426 |  |
| K.RHDVDEVC(+57.02)EM(+15.99)KPL.K | Y | 35.23 | 1642.7443 | 1.7 | 411.694 | 4 | 49.12 | 14306 | Carbamidomethylation; Oxidation (M) |
| K.NLFKDHVYK.I | Y | 33.23 | 1162.6134 | 2.1 | 582.3152 | 2 | 52.64 | 15466 |  |
| K.C(+57.02)GEDEC(+57.02)C(+57.02)IQIGN.T | Y | 32.67 | 1453.5272 | -0.6 | 727.7704 | 2 | 64.37 | 19792 | Carbamidomethylation |
| Q.IGNTLAGVC(+57.02)R.K | Y | 25.67 | 1059.5496 | 0.9 | 530.7825 | 2 | 49.59 | 14461 | Carbamidomethylation |
| R.C(+57.02)PC(+57.02)LEGL.K | Y | 25.51 | 847.3568 | 4.2 | 848.3677 | 1 | 70.26 | 22058 | Carbamidomethylation |
| N.TLAGVC(+57.02)R.K | Y | 23.21 | 775.4011 | 4.1 | 388.7094 | 2 | 22.99 | 6239 | Carbamidomethylation |
| K.IRC(+57.02)PC(+57.02)LEGL.K | Y | 21.41 | 1116.542 | 1.8 | 559.2793 | 2 | 70.26 | 22056 | Carbamidomethylation |
| K.C(+57.02)ISSK.G | N | 20.63 | 593.2843 | -67.2 | 594.2517 | 1 | 30.43 | 8493 | Carbamidomethylation |
| K.GGLVGKF.A | Y | 20.25 | 676.3907 | 1.2 | 677.3988 | 1 | 55.91 | 16577 |  |
| L.KNLFK.D | N | 19.33 | 648.3959 | 3.7 | 325.2064 | 2 | 15.01 | 3846 |  |
| V.C(+57.02)EMKPLK.N | Y | 19.21 | 904.451 | 3.9 | 453.2346 | 2 | 55.26 | 16361 | Carbamidomethylation |
| K.GGLVGKFAGK.C | Y | 16.28 | 932.5443 | 133.4 | 933.676 | 1 | 101.89 | 35236 |  |
| R.HDVDEVC(+57.02)EM(+15.99)K.P | Y | 15.73 | 1276.5063 | 4.4 | 426.5113 | 3 | 19.8 | 5283 | Carbamidomethylation; Oxidation (M) |

**Supplementary table S1:** Identified peptides by proteomic analysis in the database search against transcriptome *Nephilingis cruentata*.

| **Molecule** | **Residue** | **HADDOCK score** | **RMSD (Å)** | **Van der Waals energy** | **Electrostatic energy** | **Desolvation energy** | **Restraints violation energy** | **Z-Score** |
| --- | --- | --- | --- | --- | --- | --- | --- | --- |
| ACTX-Hvf17 | Lys^30^ | -78.4 +/- 9.0 | 8.3 +/- 0.3 | -29.6 +/- 3.6 | -220.2 +/- 43.9 | -7.7 +/- 1.9 | 29.0 +/- 7.5 | -1 |
| ACTX-Hvf17 | Arg^31^ | -84.0 +/- 1.1 | 0.7 +/- 0.2 | -36.0 +/- 0.6 | -229.7 +/- 10.0 | -4.6 +/- 2.2 | 24.7 +/- 4.8 | -2.3 |
| NcTI | Lys^31^ | -103.2 +/- 9.1 | 0.4 +/- 0.3 | -44.2 +/- 3.3 | -322.4 +/- 44.7 | 4.3 +/- 3.8 | 12.3 +/- 14.6 | -2.7 |
| NcTI | Arg^32^ | -102.7 +/- 4.1 | 0.6 +/- 0.4 | -48.5 +/- 3.8 | -297.5 +/- 12.9 | 4.2 +/- 2.9 | 10.2 +/- 9.3 | -2.6 |
| NcTI | Lys^49^ | -99.8 +/- 1.3 | 0.7 +/- 0.5 | -47.4 +/- 3.3 | -167.6 +/- 29.7 | -19.0 +/- 3.5 | 0.8 +/- 0.7 | -2.6 |
| NcTI | Lys^54^ | -103.9 +/- 4.0 | 0.5 +/- 0.3 | -55.5 +/- 4.4 | -154.2 +/- 3.2 | -17.6 +/- 2.6 | 0.7 +/- 0.8 | -1.6 |

**Supplementary table S2:** Haddock analysis results

(a)

**
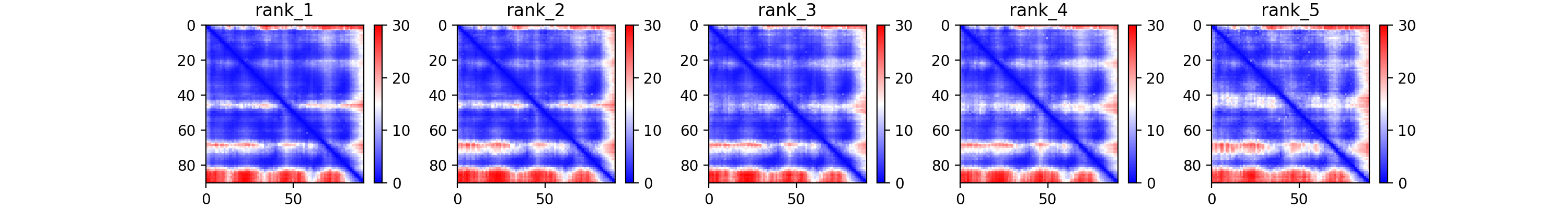
**

(b)

**
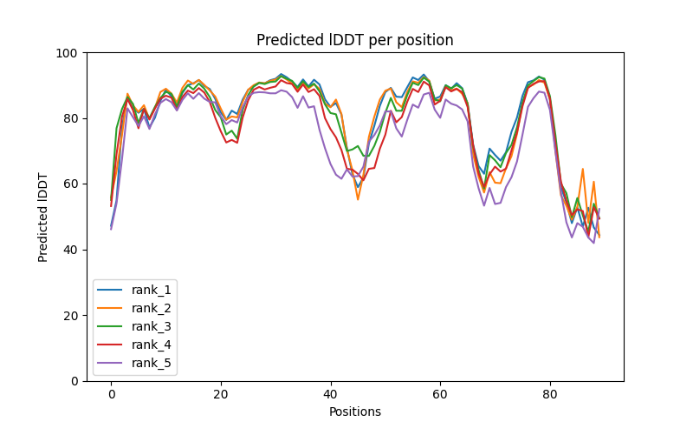
**

**Supplementary Fig. S2. Quality of modeling of Nc6834 in the ColabFold: AlphaFold2** (a): Graphic of PAE, shows the best five model in order of quality, the rank 1 was used for analysis in Fig. 6. (b): Graphic of pLDDt, representation of quality per position of amino acid residues.


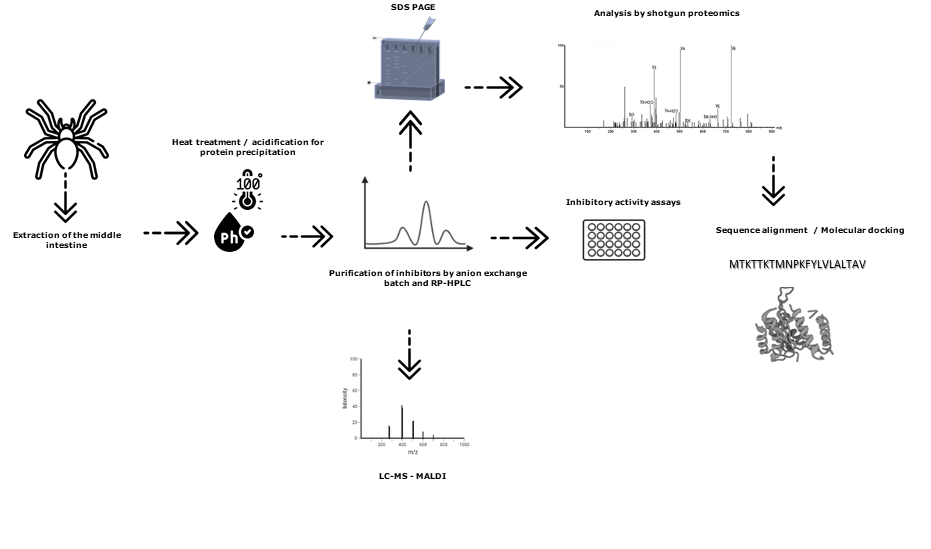


**Supplementary Fig. S3. Workflow chart of the isolation of NcTI.**
